# Gender and East–West disparities in German neurosurgical training leadership

**DOI:** 10.1007/s10143-026-04333-7

**Published:** 2026-05-23

**Authors:** Aaron Lawson McLean, Anna C. Lawson McLean, Christian Senft

**Affiliations:** Department of Neurosurgery, Jena University Hospital, Friedrich Schiller University Jena, Jena, Germany

**Keywords:** Neurosurgical training, Training program director, Gender disparities, Academic leadership, Medical education

## Abstract

Leadership in neurosurgical training is pivotal for educational quality and workforce development, yet gender and regional disparities remain poorly characterized in Germany. A nationwide cross-sectional study analyzed publicly available data from all 17 state medical chambers. Neurosurgical training program directors (TPDs) were identified, and data were collected on gender, shared training authorizations, and maximum approved training duration. Comparisons between the Old (former West) and New (former East) German states were performed using chi-square and Mann–Whitney U tests. A total of 329 TPDs were identified, of whom 26 (7.9%) were women and 303 (92.1%) men. Female representation did not differ significantly between regions (Old States 8.5% vs. New States 5.2%; *p* = 0.40). Shared training authorizations were significantly more frequent in the Old States (46.9%) than in the New States (20.7%; *p* < 0.001). Among female TPDs, 42.3% held shared authorizations, though no female-only arrangements existed. The median training duration authorized was 27 months overall (female 39 vs. male 24; *p* = 0.081). Only 37.6% of training sites offered the full 72-month curriculum required for certification, while 25.9% operated exclusively as outpatient centers. Women remain markedly underrepresented in German neurosurgical training leadership. Regional variation in shared authorizations reflects the predominance of private hospital networks in western Germany rather than differences in educational culture. Addressing these disparities will require targeted mentorship, transparent advancement pathways, and infrastructural investment to promote equitable leadership representation and standardized national training capacity.

## Introduction

Neurosurgery is among the most technically and academically demanding medical specialties, with training leaders serving as custodians of the field by setting clinical standards and mentoring future neurosurgeons. In Germany, neurosurgical training is regulated by state medical chambers (Landesärztekammern), which grant postgraduate training authority (Weiterbildungsbefugnis) predominantly to departmental chairs or senior faculty. Given that the role of the German Weiterbilder in neurosurgery, responsible for curriculum oversight, regulatory compliance, and resident mentorship, is analogous to that of training program directors (TPDs) as defined by the Accreditation Council for Graduate Medical Education (ACGME) in the United States and similar bodies in the United Kingdom and Canada, we adopt the term “TPD” to facilitate meaningful cross-national comparisons.

Despite an increasing number of women entering medicine, leadership in neurosurgery remains disproportionately male. Prior studies have noted a limited representation of women in academic leadership in neurosurgery [1–4]. For example, in Germany, while 62.5% of medical students are female as of 2019, only 13% of academic leadership positions are occupied by women [5]. Similarly, a pan-European analysis of 961 neurosurgical departments found that only 4.3% of department chairs were female, with two-thirds of European countries lacking any female leadership at all [4]. These disparities are not confined to Europe and North America. In Africa, neurosurgery remains one of the least gender-equitable surgical disciplines, with pioneering female neurosurgeons facing deeply conservative institutional environments [6]. In Asia and Australasia, while women have made notable contributions since the 1960s, some nations in the region have yet to train their first female neurosurgeon [7]. At the organizational level, a global analysis of neurosurgical societies found that women held only 29% of executive committee positions worldwide, with significant disparities in research productivity between genders [8]. Although prior studies have noted a limited representation of women in academic leadership, a significant gap persists in our understanding of how these disparities manifest within neurosurgical training leadership specifically. This underrepresentation is particularly concerning given the critical role that TPDs play in shaping educational and clinical practice standards.

This cross-sectional analysis, the first of its kind in the German neurosurgical context, aims to quantify and analyze the gender distribution and regional disparities among TPDs. By focusing on the German model, where historical distinctions between the “Old States” (traditional Western federal states) and the “New States” (former East German states) contribute to variations in institutional legacies and resource allocation, our study fills an important gap in the literature. The Old States–New States distinction remains analytically salient because over three decades after reunification, substantial structural differences persist in healthcare infrastructure, hospital ownership models, physician density, and economic capacity between the two regions [9–12]. These disparities are not merely historical but continue to shape institutional scale, resource allocation, and the conditions under which training authorizations are granted. The study provides insights into the current state of neurosurgical leadership and situates these findings within broader international trends, thereby informing efforts to promote gender equity and enhance training standards.

## Methods

We conducted a cross-sectional analysis in January 2023 by accessing publicly available data from the websites of the 17 state medical chambers across Germany’s 16 federal states (one state, North Rhine-Westphalia, maintains two historically distinct chambers). Our search focused on identifying neurosurgical TPDs, that is, board-certified neurosurgeons who have been granted postgraduate training authority to supervise and structure neurosurgical specialty training. The application process for this certification requires demonstration of extensive clinical experience, a sufficient patient caseload, and the presence of appropriate infrastructural conditions as mandated by the training regulations.

Gender was assigned using a dual approach: textual information (including names and pronouns in official biographies) was complemented by visual assessment of profile photographs. Two independent reviewers with expertise in neurosurgery and academic medical education conducted these evaluations, resolving any discrepancies by consensus.

We recorded whether training authority was organised as an individual, shared, or networked arrangement. In the German system, the postgraduate training authorisation (Weiterbildungsbefugnis) may be granted to one physician or distributed across multiple authorised trainers at the same institution or across affiliated sites. Depending on the regulatory and institutional arrangement, defined portions of the total training period may be allocated to different trainers, or training may occur within a jointly authorised structure. Thus, for a specialty requiring 72 months of training, the full authorisation may be divided across more than one trainer, such that no single individual necessarily holds the complete training mandate. These arrangements primarily reflect organisational and regulatory structures rather than a separate pedagogical model. We additionally recorded the number of affiliated training sites and the maximum authorised training duration per TPD, ranging from 6 to 72 months, with 72 months corresponding to the full required neurosurgical training period.

Additionally, we conducted a subgroup analysis comparing the Old States (Baden-Württemberg, Bavaria, Berlin, Bremen, Hamburg, Hesse, Lower Saxony, North Rhine-Westphalia, Rhineland-Palatinate, Saarland, and Schleswig-Holstein) with the New States (Brandenburg, Mecklenburg-Western Pomerania, Saxony, Saxony-Anhalt, and Thuringia). Despite its unique historical context as a city once divided into East and West, Berlin was categorized as part of the Old States due to its robust academic infrastructure, extensive research networks, and economic indicators aligning with traditional Western states [13]. Governmental and academic analyses, such as socioeconomic assessments by the German Federal Statistical Office (Destatis) and studies by the Federal Institute for Research on Building, Urban Affairs and Spatial Development (BBSR), consistently classify Berlin with the Old States. State population figures, current as of 31 December 2023, were obtained from Destatis [14].

Descriptive statistics were calculated for all variables. The Mann–Whitney U test was employed to compare median training durations between male and female TPDs (statistical significance defined as *p* < 0.05). For categorical variables such as the proportion of shared training license arrangements and the proportion of female TPDs between regions, chi-square tests were used. All statistical analyses were conducted using IBM SPSS Statistics for Windows, Version 29 (IBM Corp., Armonk, NY, USA).

## Results

Our analysis identified a total of 329 neurosurgical TPDs across Germany, of whom 26 (7.9%) were female and 303 (92.1%) male. Disaggregated state-level data are presented in Table 1. Regionally, 23 of 271 TPDs (8.5%) in the Old States and 3 of 58 (5.2%) in the New States were female. This difference was not statistically significant (χ²(1) = 0.72, *p* = 0.40). Shared training supervision arrangements were observed for 139 of 329 TPDs (42.2%). These were significantly more common in the Old States (127/271, 46.9%) than in the New States (12/58, 20.7%) (χ²(1) = 13.51, *p* < 0.001; see Table 2). Among female TPDs, 11 of 26 (42.3%) participated in shared models; however, no instance involved two women jointly sharing training authority.

**Table 1:** Distribution of neurosurgical training program directors (TPDs) and training site characteristics by federal state

Federal state	No. of TPDs	Female TPDs	Male TPDs	Shared TPD Pos.	No. of training sites	Sites Full 72 Mo.	Outpatient-only sites	Median training time (F, months)	Median training time (M, months)	State population	% of national population
Baden-Württemberg	32	3	29	18	16	10	6	18	48	11,339,260	13.4%
Bavaria	61	3	58	21	58	17	13	36	12	13,435,062	15.9%
Berlin	18	2	16	0	19	8	6	42	42	3,782,202	4.5%
Brandenburg	10	0	10	2	11	6	0	0	72	2,581,667	3.0%
Bremen	2	0	2	2	1	0	0	0	18	691,703	0.8%
Hamburg	21	1	20	9	15	2	4	48	24	1,910,160	2.3%
Hessen	28	4	24	13	20	5	6	42	24	6,420,729	7.6%
Mecklenburg-Vorpommern	13	0	13	4	9	5	3	0	72	1,629,464	1.9%
Lower Saxony	47	4	43	40	25	4	11	60	18	8,161,981	9.6%
North Rhine-Westphalia	25	2	23	6	23	12	9	48	72	18,190,422	21.5%
Rhineland-Palatinate	15	3	12	10	11	4	3	12	48	4,174,311	4.9%
Saarland	6	1	5	2	5	2	0	12	12	994,424	1.2%
Saxony	14	1	13	0	13	7	1	72	54	4,089,467	4.8%
Saxony-Anhalt	9	0	9	0	7	4	2	0	66	2,180,448	2.6%
Schleswig-Holstein	16	0	16	6	13	5	1	0	42	2,965,691	3.5%
Thuringia	12	2	10	6	9	5	1	42	72	2,122,335	2.5%
Total	329	26	303	139	255	96	66	39.0	24.0	84,669,326	100.0%

This table summarizes neurosurgical Training Program Directors (TPDs) and affiliated training site characteristics across German federal states. It includes the total number of TPDs, disaggregated by gender, and notes shared TPD positions as a proxy for collaborative leadership. Training sites are classified by their ability to offer the full 72-month program required for board certification or as outpatient-only centers. Median training durations for female and male TPDs are also shown. State population figures (as of 31 December 2023) are from the Federal Statistical Office of Germany

**Table 2:** Comparison of regional characteristics between old states and new states

Regional Classification	Total TPDs	Female TPDs (*n*, %)	Shared TPD positions (*n*, %)
Old States (West)	271	23 (8.5%)	127 (46.9%)
New States (East)	58	3 (5.2%)	12 (20.7%)
Statistical Comparison		χ²(1, *N* = 329) = 0.72, *p* = 0.40 (Female Proportions)	χ²(1, *N* = 329) = 13.51, *p* < 0.001 (Shared TPD Arrangements)

This table compares neurosurgical Training Program Directors (TPDs) between former West (“Old States”) and East (“New States”) German regions. It reports the total number of TPDs, the number and percentage of female TPDs, and the number and percentage involved in shared training license arrangements. The final row presents chi-square test results. Female representation did not differ significantly by region, whereas shared arrangements were significantly more common in the Old States

The network of training sites included 255 facilities, of which only 96 (37.6%) offered the full 72-month training program required for board certification and 66 (25.9%) operated exclusively as outpatient centers. The maximum training duration offered by individual TPDs ranged from 6 to 72 months, with an overall median of 27 months. When stratified by gender, female TPDs offered a median of 39 months of training compared with 24 months provided by their male counterparts; however, this difference was not statistically significant (Mann–Whitney U test, *p* = 0.081).

## Discussion

Importantly, our analysis encompasses the entire German neurosurgical training system, not only academic, university-attached departments but also public university hospitals, private for-profit, and private non-profit centers, capturing the full spectrum of organizational models within the German healthcare landscape and thereby enhancing the relevance of our findings for both policymakers and clinical practitioners.

### Gender disparities in neurosurgical leadership

Our findings demonstrate a pronounced underrepresentation of women among neurosurgical TPDs in Germany, with only 7.9% of all positions held by women and just 5.2% in the eastern New States. This pattern mirrors the broader gender gap observed in departmental leadership and reflects the persistent structural barriers that hinder women’s advancement from clinical qualification to senior academic or administrative roles [4, 5, 15]. Despite the near parity of male and female medical graduates, women remain significantly less likely to attain leadership positions within neurosurgery, underscoring the continued presence of a professional “glass ceiling.” This pattern is not unique to the European context. Globally, female neurosurgeons held only 29% of executive committee positions in neurosurgical societies [8], and in regions such as Africa, women remain severely underrepresented in the specialty despite increasing numbers entering medicine [6]. In Asia and Australasia, pioneering women neurosurgeons have achieved leadership roles in several countries, yet deeply conservative institutional environments in parts of the region have prevented even the training of a first female neurosurgeon in some nations [7]. The German figures thus form part of a worldwide pattern of gender inequity in neurosurgical leadership.

Within the small cohort of female TPDs, 42.3% occupy shared leadership positions; however, no case involved two women sharing the same training authorization. This indicates that while women are represented within collaborative models, their inclusion does not necessarily confer equivalent authority or decision-making power. Whether such shared arrangements facilitate genuine professional integration or instead serve to limit autonomous leadership within predominantly male hierarchies remains an open question.

Although the Weiterbildungsbefugnis—and by extension the maximum training duration—are formally determined by the regional medical chambers based on institutional infrastructure and caseload, the aggregate distribution remains instructive. Female-led departments are disproportionately represented among institutions with shorter training authorizations, suggesting that women more frequently lead smaller departments or hospitals with limited operative volume and resources. Conversely, the observed (though statistically nonsignificant) trend toward longer authorizations among some female TPDs may reflect institutional diversity rather than individual mentoring style. In this context, training duration serves as a proxy for departmental capacity and institutional privilege. Its uneven distribution across gender likely reflects underlying structural inequalities in access to large, well-resourced centers and to the career opportunities such environments afford.

### Regional variations and collaborative models

In addition to gender disparities, marked geographic variation was observed across Germany. While female representation was slightly lower in the eastern New States (5.2%) than in the western Old States (8.5%), this difference was not statistically significant. By contrast, the divergence in collaborative leadership models was both substantial and significant: 46.9% of training program directors (TPDs) in the Old States held shared training authorizations compared with only 20.7% in the New States (*p* < 0.001).

This regional imbalance likely reflects institutional organisation and ownership structure rather than intrinsic differences in educational philosophy. Shared training authorisations are a regulatory arrangement in which training authority may be distributed across multiple authorised trainers within the same institution or across affiliated sites. Depending on the institutional structure, this may involve defined portions of the overall training period being allocated to different trainers or training taking place within a jointly authorised framework. Thus, in a specialty requiring 72 months of training, the full authorisation may be divided across several training programme directors rather than being held entirely by one individual. The greater prevalence of such arrangements in western Germany may plausibly relate to the concentration of large hospital groups and multi-site provider networks, including private chains such as Helios, Asklepios, and Sana, whose organisational models distribute training responsibilities across several locations and senior physicians. On this reading, the disparity appears more likely to reflect regulatory and operational structures than any distinct pedagogical model.

Nevertheless, the structural implications remain significant. In eastern Germany, where such corporate networks are less prevalent, smaller or stand-alone hospitals may lack the infrastructural capacity necessary to qualify for full training authorizations or to support distributed models of educational governance. As a result, trainees in these settings face reduced exposure to complex or high-volume cases and may need to transfer between institutions to complete the 72-month curriculum. These transfers, often logistically and professionally burdensome, contribute to uneven access to comprehensive training and perpetuate regional inequities in workforce development. The combined effects of smaller institutional scale, lower physician density, and limited cross-institutional collaboration in the New States may therefore reinforce disparities in training continuity, mentorship, and career advancement [9–12].

### Implications for mentorship, academic culture, and training standardization

The persistent underrepresentation of women in educational leadership has far-reaching consequences for mentorship, institutional culture, and professional advancement within neurosurgery. Departmental chairs and training program directors (TPDs) occupy pivotal positions in defining curricula, setting institutional priorities, and serving as mentors for the next generation of surgeons [16]. A limited presence of women in these roles restricts mentorship opportunities for female trainees and perpetuates systemic imbalances across authorship, promotion, and academic visibility [1, 17–28].

Female neurosurgeons continue to face disproportionate structural and cultural barriers in career progression, including work–life constraints, inequitable promotion pathways, and gender-based discrimination, reported by 88% of female neurosurgeons compared with 38.1% of their male counterparts [17, 18]. These disparities mirror trends observed in North America, where women remain less likely to achieve full professorships, and are similarly evident in other surgical fields such as cardiothoracic and plastic surgery [1, 19–24]. Moreover, these patterns extend beyond the Euro-American context: in Africa, historical and structural barriers have restricted women’s entry into and advancement within neurosurgery [6], while in Asia and Australasia, progress has been uneven, with some countries yet to produce their first female neurosurgeon despite decades of specialty development [7]. Academic visibility follows the same pattern: women remain underrepresented as invited speakers and senior authors, accounting for only 7.4% of last authors in major neurosurgical journals [25–28]. Together, these data reflect an enduring structural imbalance that restricts women’s influence in shaping educational culture and professional standards.

At the same time, heterogeneity in training capacity and institutional structure complicates the standardization of German neurosurgical education. Only 37.6% of centers offer the complete 72-month curriculum required for certification, whereas approximately one quarter operate exclusively as outpatient facilities, limiting operative and inpatient exposure. This fragmented system necessitates frequent inter-institutional transfers, which impose logistical and professional burdens on trainees. Such transfers are especially common in the New States and in smaller hospitals—institutions more likely to be led by women—thereby compounding both gendered and regional inequities [29]. Ensuring equitable access to comprehensive training thus requires addressing these intersecting structural disparities within both the academic and geographic dimensions of the neurosurgical training landscape.

### Policy implications and future directions

Achieving greater equity in neurosurgical training leadership requires coordinated institutional and policy-level action. Structured mentorship programs, transparent selection procedures, and active sponsorship of female neurosurgeons should be paired with regulatory adaptations that allow smaller or peripheral departments to serve as legitimate training providers. Expanding recognition of such institutions would help counteract the concentration of full training authorizations in large, resource-rich centers where women remain underrepresented.

Parallel reforms to reduce East–West disparities should prioritize targeted investment, inter-hospital collaboration, and digital learning infrastructures that compensate for differences in caseload and institutional scale. Strengthening cooperative training networks could both broaden access to comprehensive 72-month programs and lessen the need for disruptive institutional transfers among residents.

To enhance female representation in leadership, sustained commitment is required from German neurosurgical institutions, including departments, hospitals, and medical chambers [4, 15]. These efforts should complement ongoing initiatives such as the EANS Diversity Committee but must be embedded within national systems of career development, evaluation, and promotion. Transparent advancement criteria, equitable mentorship opportunities, and standardized leadership selection processes are essential to dismantle structural barriers and ensure genuine parity of access.

Future research should incorporate institutional variables, such as hospital ownership, case volume, and accreditation level, to disentangle individual from structural determinants of training authority. Qualitative inquiry into the lived experience of training directors could further clarify how gender and institutional context interact to shape leadership trajectories. Longitudinal monitoring by professional societies would provide accountability and enable systematic evaluation of the impact of these interventions over time. Critically, the descriptive findings of the present study should be validated through a targeted survey of the identified TPDs, which would enable direct assessment of the motivations, barriers, and institutional dynamics underlying the observed gender and regional disparities, thereby moving beyond the inherent interpretive limitations of publicly available registry data.

## Limitations

This study’s findings should be interpreted in light of several limitations. Firstly, our analysis relies entirely on data collected from publicly available state medical chamber websites. The accuracy, completeness, and timeliness of this information are contingent upon the diligence of the respective chambers in maintaining their online registries; potential omissions or outdated entries cannot be fully excluded. Secondly, gender assignment was necessarily based on textual and visual cues from public profiles, resulting in a binary (male/female) classification. This methodological approach, while standard for registry-based studies, does not capture the full spectrum of gender identity or account for non-binary individuals or instances where self-identification may differ from public records. Thirdly, the cross-sectional design provides only a snapshot as of January 2023. This precludes the analysis of temporal trends in leadership representation, individual career trajectories towards TPD roles, or the causal factors underlying the observed disparities. Fourthly, while the German Weiterbilder role shares key functions with Training Program Directors in other countries, inherent differences in national healthcare systems, contextual factors, accreditation processes (e.g., ACGME), appointment procedures, and specific regulatory duties may constrain direct international comparisons, particularly with US or UK models. Finally, this quantitative analysis does not incorporate qualitative data; insights into the lived experiences, specific challenges, or perceived barriers related to leadership and gender equity from the perspectives of TPDs or trainees are beyond the scope of this study.

## Conclusion

This nationwide cross-sectional study delineates gender and regional disparities in neurosurgical training leadership across Germany. Female neurosurgeons remain markedly underrepresented, particularly in the eastern states, and shared training authorizations are substantially more prevalent in western regions—a pattern linked less to educational culture than to the regional distribution of private hospital networks. Although training durations are formally assigned by medical chambers, their uneven distribution across gender and geography likely reflects deeper structural asymmetries in institutional capacity and opportunity.

Promoting equity will require concurrent strategies: expanding infrastructural eligibility for smaller departments, supporting female mentorship pipelines, and fostering collaboration across regional and ownership boundaries. Longitudinal and mixed-methods studies are needed to clarify the mechanisms driving these disparities and to assess the impact of ongoing diversity and standardization initiatives within German neurosurgical education.

## Data Availability

The data underlying this study were obtained in January 2023 from publicly accessible webpages of the 17 German state medical chambers. Limited supporting materials, namely the extracted analytic dataset and coding scheme generated by the authors, are available from the corresponding author on reasonable request.

## Declarations

All authors meet ICMJE authorship criteria, contributed substantially to the study, revised and approved the final manuscript, and accept accountability for its content.

### Clinical trial registration

Not applicable. This was an observational analysis of publicly available data, with no patient enrollment, prospective assignment, or intervention.
